# Acarbose Decreases the Rheumatoid Arthritis Risk of Diabetic Patients and Attenuates the Incidence and Severity of Collagen-induced Arthritis in Mice

**DOI:** 10.1038/srep18288

**Published:** 2015-12-18

**Authors:** Hsin-Hua Chen, Der-Yuan Chen, Ya-Hsuan Chao, Yi-Ming Chen, Chao-Liang Wu, Kuo-Lung Lai, Ching-Heng Lin, Chi-Chen Lin

**Affiliations:** 1 Department of Medical Research, Taichung Veterans General Hospital, Taichung, Taiwan; 2 Institute of Biomedical Science and Rong Hsing Research Center for Translational Medicine, National Chung-Hsing University, Taichung, Taiwan; 3 School of Medicine, National Yang-Ming University, Taipei, Taiwan; 4 Division of Allergy, Immunology and Rheumatology, Department of Internal Medicine, Taichung Veterans General Hospital, Taichung, Taiwan; 5 School of Medicine, Chung-Shan Medical University, Taichung, Taiwan; 6 Institute of Public Health and Community Medicine Research Center, National Yang-Ming University, Taiwan; 7 Department of Medical Education, Taichung Veterans General Hospital, Taichung, Taiwan; 8 Department of Biochemistry and Molecular Biology, National Cheng Kung University Medical College, Tainan, Taiwan

## Abstract

Acarbose has been found to decrease some inflammatory parameters in diabetic patients. This study aimed to examine the influence of acarbose on rheumatoid arthritis (RA) risk in diabetes mellitus (DM) patients and on the incidence and severity of collagen-induced arthritis (CIA) in mice. In a nationwide, matched case–control study, we identified 723 incident RA cases and selected 7,230 age-, sex- and RA diagnosis date–matched controls from all newly treated DM patients. We found that use of acarbose at > 16,950 mg per year was associated with a lower RA risk (odds ratio 0.60; 95% CI, 0.41–0.89). In the CIA mouse study, acarbose was orally administered from days -7 to 38 relative to type II collagen (CII) immunization. The results revealed that acarbose at the dose of 500 mg/kg/day attenuated the incidence and severity of arthritis and the expression of proinflammatory cytokines, including TNF-α, IL-6 and IL-17 in the paw tissues. Acarbose further decreased the productions of anti-CII-IgG, IL-17 and IFN-γ by collagen-reactive lymph node cells. This work suggests that the use of acarbose decreased RA risk in DM patients and the incidence of CIA in mice. Acarbose also attenuated the severity of CIA via anti-inflammatory and immunomodulatory effects.

Rheumatoid arthritis (RA) is a common autoimmune, chronic inflammatory rheumatic disease characterized by progressive joint damage^1^,^2^. Accumulating evidence indicates that cellular and humoral immune responses to citrullinated proteins may lead to the development of RA^3^. The primary RA treatment strategy is the use of disease-modifying anti-rheumatic drugs (DMARDs), including biologic agents^4^,^5^. However, there are still some patients who show inadequate responses or are intolerance of the currently available DMARDs. Furthermore, concerns regarding high costs and increased infection risks limit the extensive application of biologic agents^6^. Therefore, there exists an unmet need to discover an inexpensive and safe new DMARD that can be widely used for RA therapy.

The alpha-glucosidase inhibitor acarbose is an inexpensive and well-tolerated drug that has been widely used to treat diabetes mellitus (DM) for more than 20 years^7^. The primary effect of this drug is to reduce post-prandial hyperglycemia^8^. The glucose-lowering effect of acarbose is better in Asian populations than in Western populations^9^, and acarbose has been found to exhibit an efficacy similar to that of metformin in China^10^. Acarbose is a pseudo-carbohydrate that competitively inhibits alpha-glucosidases in the brush border of the small intestine. This inhibition delays carbohydrate breakdown in the gut and retards sugar absorption^11^,^12^. The use of acarbose without other anti-diabetic drugs does not induce hypoglycemia^7^. In addition to its anti-diabetic effect, acarbose has been found to decrease some inflammatory parameters in diabetic patients^13^,^14^. Due to its anti-inflammatory effect, acarbose also attenuates the risks of cardiovascular disease^15^. For this reason, we hypothesized that acarbose may also possess the potential to reduce the risk of RA development or treat RA. To date, no study has investigated whether acarbose can decrease RA risk. Moreover, the anti-arthritis effects of acarbose have not been studied using animal models of RA.

Recently, the Taiwanese National Health Insurance Research Database (NHIRD) made population-based longitudinal studies feasible. We have successfully utilized the NHIRD to conduct several epidemiologic studies of RA^16^,^17^,^18^. The first part of the present study aimed to conduct a population-based, matched case-control study to examine the potential RA-protective effect of acarbose using the NHIRD. Additionally, murine collagen-induced arthritis (CIA) is a T cell–dependent, antibody-mediated autoimmune disease that is directed against cartilage type II collagen (CII)^19^. CIA has been widely used to provoke arthritis that exhibits many clinical, histological and immunopathological features of human RA^20^. The CIA model has been extensively used to investigate the therapeutic effects of new drugs for RA^21^. Therefore, the second part of this study sought to investigate the potential CIA-protective effect of acarbose and its potential anti-arthritis, anti-inflammatory and immunomodulatory effects in CIA mice. We induced CIA in DBA/1 mice via the immunization of the mice with CII in Freund’s complete adjuvant^22^,^23^. We compared the CIA incidences following CII immunization between the groups that were treated with acarbose (100 mg/kg/day or 500 mg/kg/day) or water (controls) prior to the CII immunization. The anti-arthritis effect of acarbose was examined by comparing visual scores of the paws between the acarbose-treated groups and controls. We investigated the anti-inflammatory effect of acarbose by comparing joint histologies and the proinflammatory cytokine levels in arthritic paw extracts between the acarbose-treated groups and controls using enzyme-linked immunosorbent assay (ELISA) assays. The immunomodulatory effect of acarbose was investigated by comparing the CII–specific antibodies in the serum and the proliferative responses/cytokine production in the lymph node (LN) cells between the acarbose-treated groups and controls.

## Results

### Population-based case-control study

#### Demographic data

We performed a nationwide, population-based case-control study using the NHIRD. During the period of 2001–2010, we identified 723 newly diagnosed RA cases (aged ≥ 16 years) from among all of the newly treated DM patients who received at least one year of anti-diabetic therapy. We randomly selected 7,230 DM patients by matching these RA cases with controls in terms of age, gender and RA diagnosis date. Table 1 illustrates the demographic data including gender, age, and geographic region. The female-to-male ratio was 2.5:1. The means ± the standard deviations (SD) of the ages were 61.7 ± 10.8 years for the RA cases and 61.8 ± 10.5 years for the controls. The majority of patients (94.8%) were more than 45 years old. The geographic regions from which the patients were enrolled in the National Health Insurance were classified into the following four regions: northern, central, southern and eastern. A chi-square test did not reveal any significant difference in the geographic region distributions between the RA cases and controls (p = 0.085).

### Clinical characteristics

Table 2 provides a comparison of the clinical characteristics of the RA cases and controls. Chi-square tests were used to examine the categorical variables, and the continuous variables were examined with *t*-tests; the examined variables included the use of anti-diabetic drugs and statins within one year prior to the index date, DM duration on the index date, and DM end-organ disease. The proportions of RA patients using metformin, thiazolidinediones (TZD), insulin, and sulphonylurea/meglitinide were greater than those of the controls. The RA patients exhibited longer DM durations than the controls. Moreover, a greater proportion of the RA cases had DM end-organ disease (International Classification of Diseases, 9th Revision, Clinical Modification [ICD9-CM] code 250.1-9) compared with the controls (42.6% vs. 3.8%, p < 0.001). The mean Charlson comorbidity index (CCI) was greater in the RA cases than in the controls. The RA cases exhibited a higher proportion of periodontitis (ICD9-CM: 523.3–5) than the controls. The mean ± SD days of anti-diabetic drug use and statin use during the one year prior to the index date in the RA cases and controls are provided in Supplementary Table S1 online. A *t*-test revealed that the annual drug prescription days were significantly longer for metformin, insulin, and sulphonylurea/meglitinide in the RA cases compared with the controls. However, the annual acarbose prescription days were not significantly different between the RA cases and controls.

### RA-protective effect of acarbose

Table 3 provides the multivariate adjusted odds ratios (ORs) with 95% confidence intervals (CIs) for the RA risks associated with acarbose and other variables in the newly treated DM patients. Miglitol use was not included in the variables in Table 3 because none of the RA cases used miglitol. Because this was a matched case-control study, a conditional logistic regression model was applied to calculate the ORs with the 95% CIs. A two-tailed p-value of <0.05 was considered statistically significant in the study. Therefore, a protective effect was deemed significant if the OR was less than 1 and the corresponding 95% CI did not include 1.

In the multivariate analysis, all of the variables listed in Table 3 were used as covariates to adjust the influence of each variable on the RA risk. In Model 1, the patients were categorized as acarbose users and non-users. Acarbose use regardless of a dose did not exhibit a significant protective effect, whereas, but insulin use did. Metformin use, longer DM duration, the presence of DM end-organ disease and higher CCI were statistically significant risk factors for RA. Although periodontitis and residence in the eastern geographic region also exhibited trends toward increases in RA risk, these associations did not reach statistical significance. In Model 2, acarbose use was further categorized as low- or high-dose based on the median annual cumulative dose (i.e., ≤16,950 mg, >16,950 mg). High-dose acarbose users exhibited a significantly lower risk of RA compared with the non-users (OR, 0.60; 95% CI, 0.41–0.89) but the low-dose users did not. Metformin use, longer DM duration, the presence of DM end-organ disease and higher CCI were still significantly associated with a lower RA risk. The correlations of acarbose use with the uses of the other anti-diabetic drugs and statins are summarized in Supplemental Table S2 online. A higher proportion of acarbose users concurrently used other antidiabetic drugs and statins compared with the acarbose non-users. The multivariate adjusted ORs with 95% CIs for the RA risk associated with acarbose use among the different subgroups are summarized in Supplemental Table S3 online. We conducted stratified analyses based on the following factors: (1) gender (man or woman): (2) age (≤65 years, >65 years), (3) periodontitis, (4) sulphonylurea/meglitinide use, (5) metformin use, (6) TZD use, (7) insulin use, and (8) statin use. A Wald test was used to evaluate the possible modification of the acarbose associated-RA protective effect. Although the RA-protective effect of high-dose acarbose seemed to be stronger in patients aged >65 years and in statin non-users, no significant modification effects were found.

### Animal study

#### Acarbose reduces the incidence and severity of CIA

**Figure 1:**
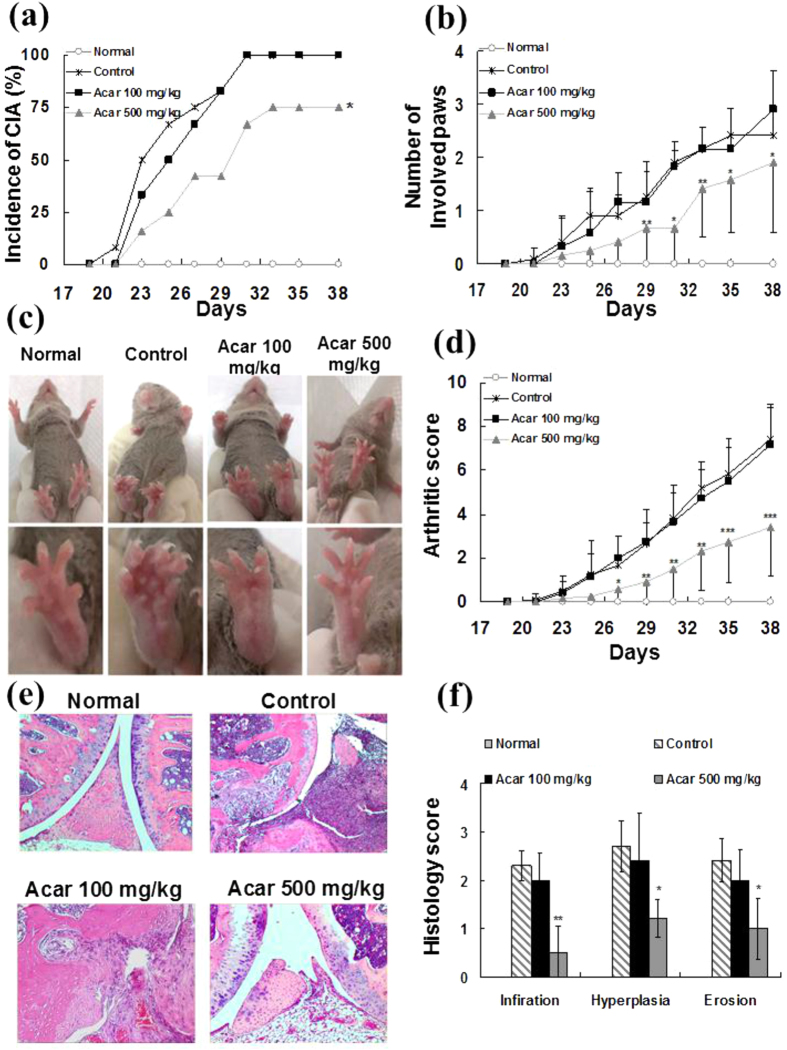
The incidence, severity, disease progression, and histopathological findings from four groups of mice (n = 12 in each group): normal mice, collagen II (CII) immunized mice treated with water (control), CII immunized mice treated with 100 mg/kg/day acarbose, and CII immunized mice treated with 500 mg/kg/day acarbose. The DBA/1 mice were dosed orally with water (control group) or acarbose (100 mg/kg/day or 500 mg/kg/day) 7 days prior to injection with collagen which continued until the end of the study (day 38). (**a**) Incidences of arthritis are represented as the percentages of mice in each group with clinical signs of arthritis (having at least one diseased paw). (**b**) The average numbers of involved paws in each mouse are shown as the mean ± standard deviation (SD). (**c**) The representative pictures of mice hind paws in 4 groups. (**d**) The average arthritis severity scores in 4 groups are shown as the mean ± SD. The severity of each paw was assessed based on a visual established semi-quantitative scoring system of 0–4: 0 = normal joint, 1 = swelling of one joint (toe/wrist/ankle/footpath), 2 = swelling of more than one joint, 3 = swelling of all joints, and 4 = bursting of the skin/dysfunction or distortion of the joint. The cumulative score for all four paws of each mouse (the maximum score was 16) was used as the arthritis score to represent the overall disease severity. (**e**) The mice were euthanized, and the joint specimen was prepared for histopathological assessment. The representative sections of the knee joint histopathologies of the 4 groups are shown (hematoxylin and eosin [H&E]; original magnification x 100). (**f**) Histological examinations for each section were scored for cell infiltrations, synovial hyperplasia, and bone/cartilage erosion from 0 to 3 (0 = absent, 1 = mild, 2 = moderate, and 3 = severe) in each group (n = 5 in each group). The results are presented as the mean ± SD. All experiments were repeated 3 times, and all data are representative of 3 independent experiments with similar results. ^*^p < 0.05; ^**^p < 0.01; ^***^p < 0.001, vs. controls.

CIA was induced in DBA/1J mice via immunization with type II collagen and Freund’s complete adjuvant as previously described^22^,^23^. We fed the mice different doses of acarbose (100 mg/kg/day or 500 mg/kg/day) or water (as a control) daily from the days -7 to 38. After the booster immunization, we monitored the mice for the occurrence of clinical signs of arthritis. As shown in Fig. 1a, a log-rank test revealed a significantly lower incidence of CIA in the group that was treated with acarbose at 500 mg/kg/day than the water-treated controls. However, acarbose at 100 mg/kg/day did not have a CIA-protective effect. Figure 1b demonstrates that the mean number of involved paws was greater in the 500 mg/kg/day acarbose-treated group than in the controls from days 29–38. Additionally, Fig. 1c,d revealed that the mean arthritic score of the control mice was significantly higher than that of the 500 mg/kg/day acarbose-treated mice during days 27–38. However, there was no significant difference in the mean arthritis scores between the 100 mg/kg/day acarbose-treated CIA mice group and the controls.

Figure 1e displays the representative histological pictures of the involved knee joints in the normal DBA/1, control and acarbose (100 mg/kg/day or 500 mg/kg/day)-treated CIA mice at day 38. Severe infiltration by inflammatory cells, synovial hyperplasia, and bone/cartilage erosion were observed in the control and 100 mg/kg/day acarbose-treated mice. In contrast, the histologic features of synovitis were less severe and extensive in the mice that were treated with acarbose at 500 mg/kg/day. The severities of cell infiltration, synovial hyperplasia and cartilage/bone erosion were semi-quantitatively graded from 0 to 3 (0, normal; 1, minimal; 2, mild; 3 severe)^24^. As shown in Fig. 1f, compared with the control mice, the average histology scores of all three of the synovitis histologic features were higher in the 500 mg/kg/day acarbose-treated mice but not in the 100 mg/kg/day acarbose-treated mice. Together, these data suggest that acarbose at 500 mg/kg/day but not at 100 mg/kg/day was effective in reducing the incidence and severity of CIA.

#### Acarbose attenuated the inflammatory responses in CIA arthritic joints

**Figure 2:**
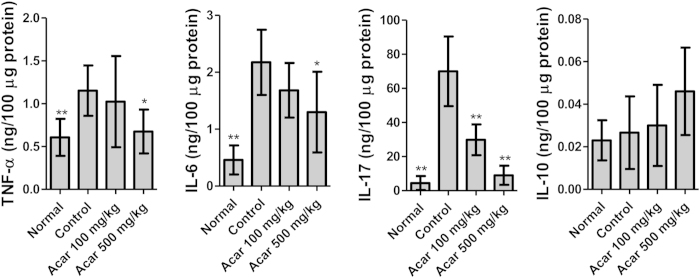
Proinflammatory cytokines in the hind paws of the four groups of mice (n = 5 in each group): normal mice, collagen II (CII)-immunized mice treated with water (control), CII immunized mice treated with 100 mg/kg/day acarbose, and CII immunized mice treated with 500 mg/kg/day acarbose. The hind paws were collected on day 38 from each group of mice (n = 5 mice/group). The paw homogenates (100 μg/well) from the individual mice were assayed in triplicate for cytokine expression by specific enzyme-linked immunosorbent assay (ELISA). The mean ± standard deviation (SD) cytokine levels for each group of mice are shown at each time point. All experiments were repeated 3 times, and all data are representative of 3 independent experiments with similar results. ^*^p < 0.05; ^**^p < 0.01, vs. controls.

CIA and RA share the characteristic feature of increased expression of pro-inflammatory cytokines, including tumor necrosis factor (TNF)-α, interleukin (IL)-6 and IL-17, in the joints^25^. Antagonism of these cytokines reduces the severity of arthritis^26^,^27^. Moreover, the anti-inflammatory cytokine IL-10 suppresses the disease processes of RA^28^. Therefore, we next investigated the effects of acarbose on the expression of TNF-α, IL-6, IL-17 and IL-10 in the arthritic paws of the CIA mice. The total cellular proteins of the diseased hind paws were extracted on day 38, and the levels of TNF-α, IL-6, IL-17 and IL-10 were measured by ELISA following standardization of the protein concentrations. Figure 2 reveals that the expression of proinflammatory cytokines, including TNF-α, IL-6, and IL-17, was significantly lower in the paws of the CIA mice treated with acarbose at 500 mg/kg/day compared with the paws of the control CIA mice. However, only IL-17 was significantly lower in the 100 mg/kg/day acarbose-treated mice than the controls. Additionally, there were no significant differences in IL-10 expression between the control group and the acarbose-treated groups (Fig. 2). Thus, these data suggested that acarbose at 500 mg/kg/day attenuates the inflammatory response in arthritic joints of CIA mice.

#### Acarbose reduced anti-CII antibody in the serum of the CIA mice

**Figure 3:**
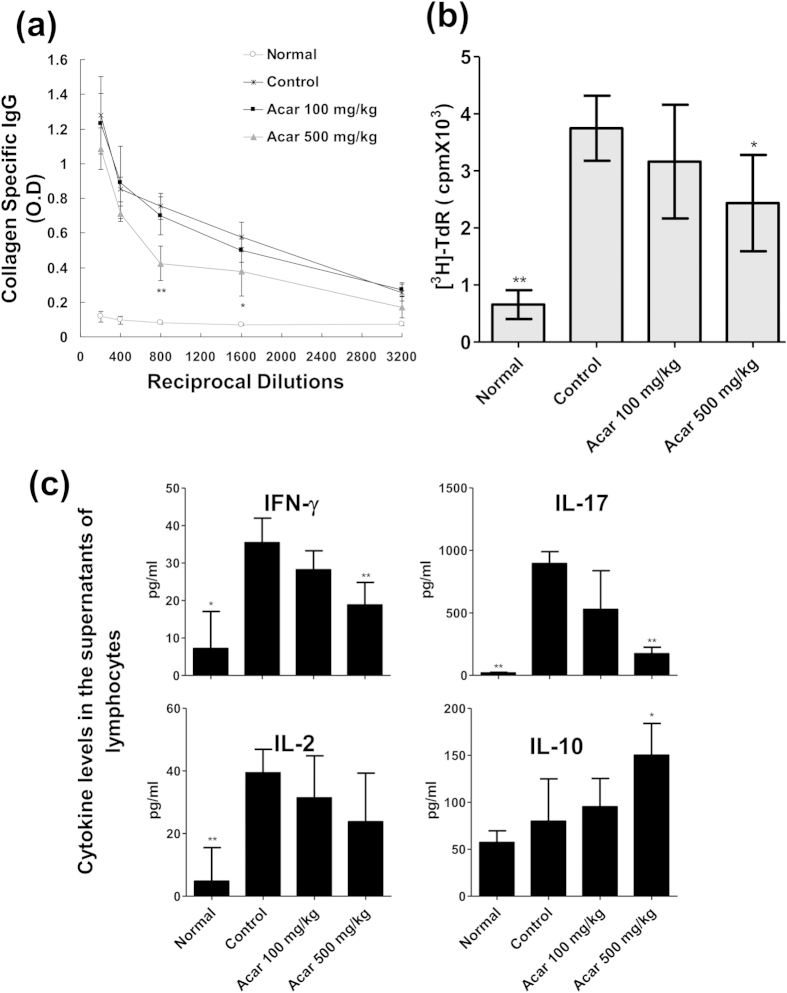
Collagen type II (CII)-specific autoantibody production in serum and *in vitro* CII-induced lymph node T cells expansion and cytokine production in the following four groups of mice (n = 5 in each group): normal mice, and collagen II-immunized mice treated with water (control), low-dose acarbose (100 mg/kg/day) mice, and high-dose (500 mg/kg/day) acarbose. (**a**) The IgG reactivity to CII in serum tested at different dilutions (in two-fold steps from 1:200 to 1:3,200) by enzyme-linked immunosorbent assay (ELISA). Serum from normal DBA/1 mice, control and acarbose (100 mg/kg/day or 500 mg/kg/day)-treated CIA mice were precipitated. CII-reactive IgG within serial dilutions of serum precipitates were determined in triplicate by ELISA. The values show the mean ± standard deviation (SD) of the optical density at 450 nm (OD450). (**b**) Inguinal lymph node cells (5 × 10^5^ cells/100 μl per well) from each group of mice were cultured in triplicate in media containing 40 μg/ml CII for 64 hr. And then there was pulsing with 1 Ci [^3^H]thymidine for another 18 hr. The cell proliferations assay was measured by the [^3^H]TdR incorporation assay. The values show the mean ± SD counts per minute (c.p.m.). (**c**) Regarding the in vitro cytokine analysis, inguinal LN cells (5 × 10^5^ cells/100 μl per well) of different mice groups were stimulated at 40 μg/ml CII under the same conditions used for the proliferation assay. After 48 h, the culture supernatants were collected, and IL-17, IFN-γ, IL-2, and IL-10 production were measured in the triplicate well by ELISA. The values revealed that the mean ± SD optical density at 450 nm (OD450). All experiments were repeated 3 times, and data are representative of 3 independent experiments with similar results. ^*^p < 0.05; ^**^p < 0.01, vs. controls.

To further evaluate the immunomodulatory effect of acarbose, we examined the levels of CII-specific IgG Ab in CIA mice. The various dilution of serum (in two fold steps from 1:200 to 1:3,200) from 4 groups of mice bled on day 38 were measured via ELISA assay. As shown in Fig. 3a, the serum CII-specific IgG level in the 500 mg/kg/day acarbose-treated mice was significantly lower than that in the controls at the dilutions of 1/800 to 1/1,600. However, the IgG levels were not different between the 100 mg/kg/day-treated group and control group at any of the dilutions.

#### Acarbose modulated the CII-specific T cell response in CIA

We collected LN cells (5 × 10^5^ cells/100 μl per well) from the arthritic hind limbs on day 38. In the presence of CII antigen, the *in vitro* proliferative responses and cytokine production were examined by [^3^H]-thymidine incorporation and ELISA, respectively. As shown in Fig. 3b, the mice treated with acarbose at the dose of 500 mg/kg/day but not those treated with 100 mg/kg/day exhibited less CII-specific lymphocyte proliferation than the controls. Compared with the controls, the levels of IFN-γ and IL-17 in the supernatants of the lymphocytes were significantly lower in the 500 mg/kg/day acarbose-treated mice but not in the 100 mg/kg/day acarbose-treated mice (Fig. 3c). The IL-10 levels in the lymphocyte supernatants were higher in the 500 mg/kg/day acarbose-treated mice than in the controls. There was no significant difference in the IL-2 levels between the controls and acarbose-treated groups.

## Discussion

In the population-based study, we found that acarbose use with an annual cumulative dose of more than 16,950 mg (daily average dose 46.4 mg) decrease the risk of RA by 40% after adjusting for potential confounders. Notably, this RA-protective effect was not significantly different between the various subgroups that were stratified based on the patients’ characteristics, other anti-diabetic drug use or statin use. In the CIA study in the mice, we confirmed the arthritis-protective effect of acarbose at the dose of 500 mg/kg/day but not 100 mg/kg/day as evidenced by the decreased incidence and severity of arthritis following CII immunization. Acarbose at 500 mg/kg/day also exerted anti-inflammatory and immunomodulatory effects as revealed by the suppression of the expression of pro-inflammatory cytokines, including TNF-α, IL-6, and IL-17, in the arthritic paws, the level of CII-specific IgG autoantibodies, and the CII-specific CD4 + T cell responses. Consistent with the results of our animal study, Derosa *et al.* reported that acarbose decreases inflammatory markers, including IL-6, in Caucasian type 2 diabetic patients^13^. Additionally, Benli *et al.* found that acarbose reduces serum levels of inflammatory cytokines, including TNF-α, in Chinese type 2 diabetes patients^14^. However, no previous studies have used a CIA mouse model to investigate the anti-inflammatory effects of acarbose. Taken together, the results of our population-based human study and CIA mouse study indicate that the use of acarbose at more than a specific dose may have the potential to treat RA by modulating both cellular and humoral immune responses to antigens. Hence, it is reasonable to suggest acarbose as a candidate therapy for ameliorating RA activity.

Considering the possible mechanisms of the anti-inflammatory and immunomodulatory effects, it is important to note that acarbose is poorly absorbed^12^. Therefore, its anti-inflammatory and immunomodulatory effects are most likely attributable to extra-glycemic effects on the gut. First, acarbose has been demonstrated to regulate gut hormones, e.g., decreasing glucose-dependent insulinotropic polypeptide (GIP) and increasing glucagon-like peptide-1 (GLP-1) in the serum^29^,^30^,^31^,^32^. Previous studies have discovered that GIP has a pro-inflammatory effects *in vivo*^33^,^34^, whereas GLP-1 has anti-inflammatory effects *in vitro* and *in vivo*^35^,^36^,^37^. Recently, Chen *et al.* summarized the literature regarding the effects of gut hormones on inflammation, appetite and energy balance and suggested that gut hormones may play roles in RA^38^. Second, acarbose may interact with gut microbiota, which are possible extra-articular triggers of RA^39^. An *in vitro* study indicated that acarbose decreased the fraction of luminal *Prevotella*^40^, which has been reported to be expanded in the intestine of most DMARD-naïve incident RA patients^41^. A recent study also found that acarbose increased the fecal bifidobacteria content of patients with type 2 DM^42^. Additionally, probiotic *Bifidobacterium breve* treatment has recently been reported to induce IL-10-producing Tr1 cells in the colon via intestinal dendritic cells (DCs) and to reduce intestinal inflammation^43^. Third, the unabsorbed acarbose may have an effect on the intestinal immune system. Zhang *et al.* showed that acarbose can suppress proinflammatory cytokine expression in the gut by activating the miRNAs miR-10a-5p and miR-664 in diabetic rats^44^.

Compared with our previous case-control study on RA risk^18^, the patients recruited in the present population-based study were older because only DM patients were eligible for this study. Although periodontitis and eastern geographic region residence also exhibited trends toward an increase RA risk, these associations did not reach statistical significance as we had reported previously^18^. A secondary finding of this population-based study is that insulin use is also associated with a lower risk of RA. This result could be explained by the stated modulatory effect of insulin on toll-like receptors and inflammatory processes^45^,^46^. Another secondary finding is that metformin is associated with an increased RA risk. This finding was unexpected because previous studies have found metformin to be associated with decreased levels of inflammatory markers^47^,^48^. However, hyperhomocysteinemia, which is a potential side effect of metformin^49^, has been found to be associated with elevated levels of immune activation markers in RA patients^50^. However, further cohort studies and animal studies are warranted to confirm the influences of insulin and metformin on the risk of RA.

This study has some strengths. To the best of our knowledge, this study is the first to investigate the potential RA-protective and therapeutic effects of acarbose concurrently using population-based claims data and a CIA mouse model. The strength of the population-based study is that it minimized selection bias. The advantage of the CIA study is that it explored the *in vivo* anti-arthritis, anti-inflammatory and immunomodularty effects.

Some limitations of this research must be considered. First, although the BNHI regularly checks the quality of the claims to improve the accuracy of diagnoses, we cannot exclude the possibilities of miscoding- or misclassification-related bias. However, the diagnoses of RA were likely accurate in this study because a strict validation process that was conducted by reviewing radiographic and laboratory data was required prior to the issuing of a catastrophic illness certificate for RA patients. Second, the NHIRD lacked information regarding individual smoking status, which is potential confounder of RA. However, the non-differential misclassification bias related to smoking status would have biased the results toward the null. Third, the lack of laboratory data, such as serum glucose levels and glycated hemoglobin, in the NHIRD precluded the possibility of further adjustment via regression analyses. Fourth, the results of this population-based study in Taiwan might not be generalizable to other ethnic population.

## Conclusion

We found that the oral administration of acarbose reduces the risk of RA development in DM patients and attenuates the incidence and severity of CIA in mice. Acarbose also decreased the production of CII-specific antibody production in serum and Th1/Th17-related proinflammatory cytokines in LN and increased the anti-inflammatory cytokine IL-10. These findings provide evidence favoring the anti-inflammatory effects and immumomodulatory effects of acarbose. Although our data support acarbose to be a potential DMARD for RA, future randomized clinical trials are needed to prove the efficacy of acarbose in the relief of RA activity.

## Methods

### Population-based case-control study

#### Ethics statement

The Ethics Committee for Clinical Research of Taichung Veterans General Hospital approved this study protocol. The methods were performed in accordance with the approved guidelines. Written informed consent was not obtained because all personally identifying information was eliminated from the dataset prior to analysis.

### Data source

This nationwide, population-based, matched case–control study was conducted using 1999–2011 claims data from Taiwan’s National Health Insurance Research Database (NHIRD). The NHIRD comprises comprehensive encrypted healthcare data from more than 98% of Taiwan’s population that was released for research purposes. The NHIRD randomly selected one million enrollees in 2000 to form a representative database (LHID2000). The datasets used in this study consisted of claims of ambulatory care, inpatient services, enrollment files, dental services, prescriptions, and the NHI catastrophic illness files, all from 1999–2011. The NHI catastrophic illness data enrolled patients with devastating diseases, such as several potentially disabling autoimmune diseases, including RA. The medical charts of those who applied for catastrophic illness registration were reviewed by the Bureau of National Health Insurance (BNHI) routinely to validate the diagnoses. The RA diagnoses were verified by the American College of Rheumatology RA classification criteria (1987)^51^. Although the dataset lacked radiographic and laboratory data, the accuracy of coding has improved due to regular audits by the BNHI^52^.

### Study samples

#### DM subjects

We defined the DM subjects as those who had initial DM diagnoses (ICD9-CM code 250.x) after January 1, 2000 with the concurrent prescriptions of any anti-diabetes medication for ≥28 days. The time of the initial anti-diabetes drug prescription was selected as the DM diagnosis date used to measure DM duration.

#### RA cases

We identified 24,429 newly diagnosed RA patients with a catastrophic illness certificate (aged ≥ 16 years at RA diagnosis) during 2001–2010. Because the lag time from the onset of symptoms to rheumatologist referral may have been up to one year^53^, we excluded those who developed RA within one year after beginning anti-diabetes treatment to minimize the possibility of reverse causality. From these RA patients, we identified a total of 723 patients who had DM histories of at least one year before the first date of RA diagnosis (index date) as the RA cases. The index date was used to calculate the patients’ ages (from birthday to the index date), DM duration, and to define the period of comorbidities and drug use.

#### Non-RA controls

The non-RA controls were selected from enrollees in the LHID2000. Matching RA cases (10 for every case) for age at RA diagnosis (16–25, 26–35, 36–45, 46–55, 56–65, >65 years), sex and the year of the index date were randomly extracted to arrive at 7,230 DM subjects as non-RA controls with DM durations of at least one year and no previous histories of RA diagnoses during 1999–2011. We selected the date of the first ambulatory visit as the index date for the controls.

### Use of anti-diabetic drugs

Based on prescription records in the NHIRD, the patients who used acarbose, metformin, TZD, insulin or sulphonylurea/meglitinide for 28 days or more within one year prior to the index date were classified as acarbose users, metformin users, TZD users, insulin users or sulphonylurea/meglitinide users, respectively. We categorized acarbose users as high- or low-dose acarbose users according to the median annual cumulative dose (i.e., ≤16,950 mg, >16,950 mg).

### Potential confounders

Geographic region, DM duration and DM end-organ disease (ICD9-CM codes 250.1–9) were included as potential confounders. The geographic region was selected because it was found to be associated with RA risk in our previous study^18^. The geographic regions were classified as northern, central, southern and eastern regions, according to the residential area when the patients were enrolled in the NHIRD. We used the DM duration and DM end-organ disease as proxies for the DM severity. DM duration was defined as the period from the date of initiating anti-diabetic drugs to the index date. Patients with ≥ two ambulatory visits with DM end-organ disease diagnoses (ICD9-CM codes 250.1–9) during the year prior to the index date were classified as DM end-organ disease subjects. Other potential confounders included CCI as adapted by Deyo *et al.*^54^, periodontitis as defined in our previous study^18^, and the use of statin drugs for ≥28 days within the year prior to the index date. Periodontitis was selected because it has been found to increase the risk of RA^17^,^18^. Statins were chosen as a potential confounder because their persistent use has been reported to reduce the RA risk^55^. We defined the presence of the comorbidities using the calculated CCI as defined by at least two ambulatory visits with the corresponding ICD9-CM diagnoses during the one year before the index date.

### Animal study

#### Ethics statement

The Institutional Animal Care and Use Committee (IACUC) of National Chung Hsing University approved the experimental procedures (approved protocol no. NCHU-IACUC-104-027). The methods were performed in accordance with the approved guidelines.

### Mice

The development of CIA is strain-dependent; the H-2^q^ and H-2^r^ haplotypes exhibit the greatest degrees of susceptibility^56^. The DBA/1 strain (H-2^q^) is the most commonly used strain in the CIA model for pre-clinical testing of potential anti-arthritic drugs^22^. Male DBA/1 mice at the age of 8–10 weeks were purchased from The Jackson Laboratories (Bar Harbor, ME, USA). All mice were housed in specific pathogen-free condition in our facility.

### Induction of CIA

CIA was performed according to a previously described method with slight modifications^57^. In brief, chick collagen type II (CII, Chondrex Inc, Redmond, WA, USA) was dissolved overnight at 4 °C in cold 50 mM acetic acid to a concentration of 4 mg/ml and added dropwise to emulsify an equal volume of Freund’s complete adjuvant (CFA, Sigma-Aldrich, St. Louis, Mo, USA) containing 4 mg/ml of *M. tuberculosis* strain H37RA (Difco Laboratories Detroit, Michigan, USA). Experimental male DBA/1 mice were immunized intradermally at the base of the tail with 100 μl of emulsified chicken CII on day 0 and boosted 3 weeks later with the same agent and observed for up to 38 days post-primary immunization for clinical symptoms of arthritis.

### Acarbose treatment

To investigate the preventive effects of acarbose (Alfa Asesar, Bona street, Ward Hill, MA, USA) in the CIA in mice, DBA/1 mice were randomly allocated into the following 3 groups (n = 12 in each group): a water-treated control group; a high-dose (500 mg/kg/day) acarbose-treated group; and a low-dose (100 mg/kg/day) acarbose-treated group. Water or acarbose were orally administered beginning 7 days before collagen immunization (day -7). At day 38 after immunization, the majority of the control mice had at least two out of four paws reaching the maximum arthritis score of 4 and had to be euthanized according to the approved ethical guidelines.

### Clinical assessment of arthritis

We defined the development of CIA as the presence of at least one diseased paw^58^. The incidences of CIA between the acarbose-treated groups (100 mg/kg/day or 500 mg/kg/day) and controls were compared using the Kaplan-Meier method (survival analysis), and the significance of the difference was tested using the log-rank test. In the CIA model, the disease usually begins in one paw and then spread to the other paws, which indicated disease progression. We first counted the numbers of involved paws. Then, we quantified the severity of arthritis in each involved paw using an established visual semi-quantitative scoring system ranging from 0–4 per paw as previously described^59^. The arthritis scores of all of the involved paws of each mouse (maximum possible score 16) were used to represent the overall disease severity and progression as follows: 0 = normal joint, 1 = swelling of one joint (toe/wrist/ankle/footpath), 2 = swelling of more than one joint, 3 = swelling of all joints, and 4 = bursting of the skin/dysfunction or distortion of the joint. The clinical assessments were scored by two independent observers unaware of the mouse identities. Finally, we compared the mean numbers of diseased paws and the mean arthritis scores between the acarbose-treated group (100 mg/kg/day or 500 mg/kg/day) and the water-treated controls every other day from day 21 to day 38.

### Histopathological analyses

To further evaluate the histological changes in the arthritic joints, the mice were euthanized at the end of the experiment (day 38). Representative knee joints were harvested from five randomly selected mice in each group (i.e., the normal DBA/1 mice, the water-treated CIA control mice, the 100 mg/kg/day acarbose-treated CIA mice, and the 500 mg/kg/day acarbose-treated CIA mice). Next, the joint tissues were fixed, decalcified, and embedded in paraffin. Each joint section (5 μM) was stained with hematoxylin and eosin (H&E) prior to observation by light microscopy according to methods published in the previous study^60^. Semi-quantitative histological scoring was performed to grade the cell infiltration, synovial hyperplasia, and cartilage/bone erosion based on a previously described system that was modified as follows: 0 = absent, 1 = mild, 2 = moderate, and 3 = severe^24^. Next, we compared the average scores for each synovitis histologic feature between the high-dose and low-dose acarbose-treated groups and the control group.

### Measurement of the cytokine concentrations in arthritic hind paws

On day 38, the hind paws were removed from the normal DBA/1 mice, control and acarbose-treated (100 mg/kg/group or 500 mg/kg/group) CIA mice (n = 5 mice/group) and then frozen in liquid nitrogen. Before use, the frozen hind paws were thawed and cut into small pieces and resuspended in 1 ml phosphate buffered saline (PBS, pH 7.4) containing a 1x complete protease inhibitor cocktail tablet (Roche Diagonostic GmbH, Mannheim, Germany) and homogenized by shaking with 1.5-mm glass beads in a Mini-BeadBeater (BioSpec, Bartlesville, OK, USA) at 4,800 oscillations/min for 3 min. The tissue homogenates were centrifuged for 12000 x *g* for 15 min and collected and diluted to 1 mg tissue protein per ml (1 mg/ml) in homogenization buffer (PBS, pH 7.4, containing a protease inhibitor cocktail) as the stock solution. Total protein was determined by the micro bicinchoninic acid assay (BCA; Thermo scientific, Rockford, USA). TNF-α, IL-6, IL-17 and IL-10 cytokines quantifications in the 100 μl supernatants (total protein 100 μg) were determined by ELISA according the manufacturer’s instructions (PeproTech, Rocky Hill, NJ, USA). The concentrations of cytokines are expressed in ng/100 μg of total protein.

### Analysis of anti-collagen type II IgG antibody production in serum

On day 38, blood samples drawn from the normal DBA/1 mice, control, or acarbose (100 mg/kg/day or 500 mg/kg/day)-treated CIA mice (n = 5 mice/group) were placed into tubes containing acid-citrate dextrose by retro-orbital sinus puncture and allowed to sit for one hour at room temperature to clot. Next, the serum was pooled and prepared by centrifugation of the blood at 1,000 x *g* for 10 minutes at RT. The CII-specific IgG in the serum was analyzed by the ELISA method. Briefly, each well of the ELISA 96-well microtiter plate (Nalge Nunc International, Thermo Fisher Scientific, NY, USA) coated with type II chicken collagen at 10 μg/mL in Tris-buffered saline (TBS) buffer (50 mM TrisHCl [GMbiolab Co, Taichung City, Taiwan], pH7.4, 150 mM NaCl [UR, New Taipei City, Taiwan]) overnight at 4 °C, followed by a blocking step with TBS buffer plus 0.05% (v/v) Tween 20 (Sigma-Aldrich, St. Louis, Mo, USA; TBS/Tween-20, pH 7.4) containing 3% bovine serum albumin (BSA, Sigma-Aldrich, St. Louis, Mo, USA) for 1 h at room temperature. After washing 3 times with phosphate buffered saline (PBS) plus 0.05% Tween 20 (PBS/Tween-20, pH 7.4), various serum dilutions (in two-fold steps from 1:200 to 1:3,200) in PBS/Tween-20 were then applied to the wells at 4 °C overnight, and the anti-CII antibodies were allowed to bind to the antigen. The wells were then washed with PBS/Tween-20 extensively, and the HRP-conjugated sheep anti-mouse IgG (Jackson Immunoresearch Laboratories, West Baltimore Pike, West Grove, PA, USA; diluted at 1:5,000 in PBS/Tween-20) was applied, unbound antibody was washed with PBS/Tween-20, and the plate was developed using TMB (3,3′,5,5′-tetramethylbenzidine) substrate (ebioscience Inc, San diego, CA) and the reaction was stopped with 4.5 N H_2_SO_4_. The optical density (OD) was determined at 450 nm with an ELISA reader (Tecan Sunrise, Durham, NC, USA).

### T cell proliferation and cytokine production in lymph nodes

The mice were euthanized on day 38; the inguinal LNs of the hind limbs were collected from the normal DBA/1 mice, controls and acarbose (100 mg/kg/day or 500 mg/kg/day)-treated CIA mice (n = 5 mice/group). Single cell suspensions of LN cells were prepared by gentle mechanical disaggregation through 200 micron-mesh stainless steel gauze and resuspended in RPMI-1640 (Life technologies corporation, Grand Island, NY, USA) supplemented with fetal calf serum (Life technologies corporation, Grand Island, NY, USA) (10%), HEPES (Sigma-Aldrich, St. Louis, Mo, USA) (10 mM), sodium pyruvate (Life technologies corporation, Grand Island, NY, USA) (1 mM), 2-mercaptoethanol (Amresco, Solon lind Pkwy Solon, Ohio, USA) (50 mM), L-glutamine (Sigma-Aldrich, St. Louis, Mo, USA) (1%) and 100 units/ml of penicillin/streptomycin (Sigma-Aldrich, St. Louis, Mo, USA) (100 units/ml) and cultured in triplicate in 96-well flat-bottomed plates (5 × 10^5^ cells/100 μl per well) with 40 μg/ml chicken CII for 64 h at 37 °C under 5% CO2 and then pulsed with 1 Ci [^3^H]thymidine (Perkin-Elmer, Boston, MA, USA) per well for another 18 h. When the cells proliferated, [^3^H]TdR was incorporated into the new strands of chromosomal DNA that were synthesized. The more cell divisions (or the higher the proliferation rate), the more radioactivity that was incorporated into the DNA. The radioactivity of the incorporated [^3^H]TdR was counted using a Matrix 9600 direct ionization beta counter (Packard Instrument, Meridian CT, USA). The results are expressed as the mean counts per minute (cpm). For the *in vitro* cytokine analysis, the inguinal LN cells (5 × 10^5^ cells/100 μl per well) of the mice of different groups (n = 5 mice/group) were stimulated with 40 μg/ml CII under the same conditions used for the proliferation assay. After 48 h, the culture supernatants were collected, and the IL-17, IFN-γ, IL-2, and IL-10 productions were measured in triplicate using the ELISA Kits for mouse IFN-γ, IL-17, IL-2 and IL-10 according to manufacturer’s instructions (PeproTech, Rocky Hill, NJ, USA).

### Statistical analysis

For the population-based case–control study, we compared the baseline characteristics between the RA cases and controls using a *t*-test for continuous variables and a chi-square or Fisher’s exact test for categorical variables. After adjusting for potential confounders, including other anti-diabetic drug use, DM duration (each incremental year), DM end-organ disease, CCI (each incremental score) and geographic region, conditional logistic regression was used to estimate the effect of acarbose on RA risk as shown by the ORs with 95% CIs. The significance of the modifications of acarbose associated with the RA-protective effect by gender, age, periodontitis, use of other anti-diabetic drug and statin use were examined using the Wald test to calculate the p-value of the coefficient associated with the product of the indicators of acarbose use and the subgroup. All statistical calculations in the population-based study were performed using SPSS version 18.0 for Windows (SPSS, Inc., Chicago, IL, USA). For the animal study, the Kaplan-Meier method was used to analyze the incidence of CIA in the acarbose-treated groups (100 mg/kg/day and 500 mg/kg/day) and controls. Moreover, the significance of the differences were tested with log-rank tests. To compare the continuous variables between the control group and the acarbose group, Mann-Whitney *U*-tests were conducted. All statistical analyses in the animal study were conducted using GraphPad Prism (version 5 for Windows; GraphPad Software, San Diego, CA, USA). A two-tailed p-value of <0.05 was considered statistically significant for all analyses in both the human and animal studies.

## Additional Information

**How to cite this article**: Chen, H.-H. *et al.* Acarbose Decreases the Rheumatoid Arthritis Risk of Diabetic Patients and Attenuates the Incidence and Severity of Collagen-induced Arthritis in Mice. *Sci. Rep.* **5**, 18288; doi: 10.1038/srep18288 (2015).

## Supplementary Material

Supplementary Information

## Figures and Tables

**Table 1:** Demographic data of RA cases and matched controls.

	RA cases (n = 723)		Controls (n = 7,230)	
	Number	%	Number	%
Gender				
Female	517	71.5	5,170	71.5
Male	206	28.5	2,060	28.5
Age, years (mean ± SD)	61.7 ± 10.8		61.8 ± 10.5	
Age groups, years				
16–25	2	0.3	20	0.3
26–35	5	0.7	50	0.7
36–45	31	4.3	310	4.3
46–55	165	22.8	1,650	22.8
56–65	247	34.2	2,470	34.2
>65	273	37.8	2,730	37.8
Geographic region				
Northern	301	41.6	3,261	44.5
Central	155	21.4	1,680	23.2
Southern	247	32.7	2,173	30.1
Eastern	20	2.8	161	2.2

Abbreviations: RA, rheumatoid arthritis; SD, standard deviation. The Chi-square test was used to compare the proportions of categorical variables between RA cases and controls. The *t*-test was used to compare the continuous variables between RA cases and controls. A P-value of <0.05 was considered statistically significant.

**Table 2:** Clinical characteristics of RA cases and controls.

	RA cases (n = 723)		Controls (n = 7,230)		P-value
	Number	%	Number	%	
Anti-diabetic drug user*	517	71.5	5,170	71.5	
α-Glucosidase inhibitors					
Acarbose					0.156
Non-user	655	90.6	6,632	91.7	
Low-dose user	40	5.5	293	4.1	
High-dose user	28	3.6	305	4.2	
Miglitol	0	0.0	8	0.1	1.000
Sulphonylurea/meglitinide user	363	50.2	3,083	42.6	<0.001
Metformin user	447	61.8	4,071	56.3	0.004
TZD user	70	9.7	544	7.5	0.038
Insulin user	46	6.4	191	2.6	<0.001
Statin*	185	25.6	1,762	24.4	0.468
DM duration, years (mean ± SD)	3.9 ± 2.2		2.6 ± 1.8		<0.001
DM end-organ disease (ICD9-CM: 250.1–9)*	308	42.6	278	3.8	<0.001
Charlson comorbidity index* (mean ± SD)	4.7 ± 2.5		0.5 ± 1.7		<0.001
Periodontitis*^,^† (ICD9-CM: 523.3–5)	137	18.9	1,333	18.4	0.735

Abbreviations: RA, rheumatoid arthritis; TZD, Thiazolidinediones; DM, diabetes mellitus; SD, standard deviation; ICD9-CM, International Classification of Diseases, 9th Revision, Clinical Modification.

Acarbose use was further categorized as low- or high-dose based on the median annual cumulative dose (i.e., ≤16,950 mg, >16,950 mg).

^*^Data were collected during the one year before the index date.

^†^Patients received antibiotic treatment, or periodontal treatment other than dental scaling, or dental scaling more than twice per year. The Chi-square test was used to compare the proportions of categorical variables between RA cases and controls. The *t*-test was used to compare the continuous variables between RA cases and controls. A p-value of <0.05 was considered statistically significant.

**Table 3:** Multivariate adjusted ORs (95% CIs) for RA risk associated with acarbose and other variables in newly treated DM patients.

Variable	Model 1	Model 2
Anti-diabetic drug users*		
Acarbose user		
No	1	1
Yes	0.79 (0.61–1.03)*	
Low-dose user	–	1.00 (0.72–1.39)
High-dose user	–	0.60 (0.41–0.89)
Sulphonylurea/meglitinide user		
No	1	1
Yes	1.07 (0.91–1.25)	1.08 (0.92–1.26)
Metformin user		
No	1	1
Yes	1.27 (1.08–1.50)	1.26 (1.06–1.49)
TZD user		
No	1	1
Yes	0.96 (0.74–1.25)	0.96 (0.74–1.24)
Insulin user		
No	1	1
Yes	0.64 (0.46–0.89)	0.62 (0.45–0.87)
Statin user		
No	1	1
Yes	1.14 (0.96–1.35)	1.14 (0.96–1.36)
DM duration, each incremental year	1.22 (1.18–1.26)	1.22 (1.18–1.27)
DM end-organ disease (ICD9-CM: 250.1–9)	2.66 (2.22–3.18)	2.67 (2.23–3.19)
No		
Yes		
Charlson comorbidity index, each incremental score	1.28 (1.26–1.31)	1.28 (1.26–1.31)
Geographic region		
Northern	1	1
Central	0.97 (0.80–1.19)	0.97 (0.79–1.18)
Southern	0.98 (0.82–1.17)	0.98 (0.82–1.17)
Eastern	1.18 (0.74–1.89)	1.18 (0.73–1.88)
Periodontitis		
No	1	1
Yes	1.11 (0.91–1.35)	1.11 (0.91–1.35)

Abbreviations: RA, rheumatoid arthritis; DM, diabetes mellitus; TZD, thiazolidinediones; ICD9-CM, International Classification of Diseases, 9th Revision, Clinical Modification. Conditional logistic regression model was used to conduct the analyses, and all variables listed in the table were adjusted. In model 1, acarbose user was categorized as non-user and user. In model 2, acarbose use was further categorized as low- or high-dose based on the median annual cumulative dose (i.e., ≤16,950 mg, >16,950 mg).

^*^P = 0.079.
