# Supplementary material for: Acarbose Decreases the Rheumatoid Arthritis Risk of Diabetic Patients and Attenuates the Incidence and Severity of Collagen-induced Arthritis in Mice
Source: Sci Rep. 2015 Dec 18;5:18288. doi: 10.1038/srep18288 (PMC4683371; doi:10.1038/srep18288)
Supplement: Supplementary Information [file srep18288-s1.pdf]

Acarbose Decreases the Rheumatoid Arthritis Risk of Diabetic Patients and Attenuates the Incidence and Severity of Collagen-induced Arthritis in Mice

Authors: Chi-Chen Lin, Der-Yuan Chen, Ya-Hsuan Chao, Yi-Ming Chen, Chao-Liang Wu, Kuo-Lung Lai, Ching-Heng Lin, Hsin-Hua Chen*

Supplementary materials

Supplemental Table S1. The mean ± SD days of anti-diabetic drug use and statin use during the one year before the index date in RA cases and controls

	RA cases (n = 723)	Controls (n = 7,230)	P-value
Anti-diabetic drug use			
α-Glucosidase inhibitors			
Acarbose	14.8 ± 60.0	15.4 ± 62.8	0.806
Miglitol	Not use	0.1 ± 4.1	0.451
Metformin	142.8 ± 149.2	128.5 ± 147.2	0.013
TZD	19.4 ± 70.5	14.3 ± 61.3	0.062
Insulin	15.7 ± 76.5	5.9 ± 46.2	0.001
Sulphonylurea/meglitinide	111.0 ± 144.7	93.9 ± 138.0	0.002
Statin	44.9 ± 97.9	40.4 ± 91.3	0.237

Abbreviations: SD, standard deviation; RA, rheumatoid arthritis; TZD, thiazolidinediones.

A *t*-test was used to conduct the analyses.

Supplemental Table S2. Use of other anti-diabetic drugs and statin in high-dose acarbose users, low-dose acarbose users and non-users

	High-dose acarbose users (n = 333)		Low-dose acarbose users (n = 333)		Non-users (n = 7,287)		P-value
	Number	%	Number	%	Number	%	
Sulphonylurea/ meglitinide	170	51.1	189	56.8	3,087	42.4	<0.001
Metformin	210	63.1	254	76.3	4,054	55.6	<0.001
TZD	64	19.2	56	16.8	494	6.8	<0.001
Insulin	23	6.9	26	7.8	188	2.6	<0.001
Statin	124	37.2	117	35.1	1,706	23.4	<0.001

Abbreviation: TZD, thiazolidinediones.

Acarbose use was further categorized as high- or low-dose based on the median annual dose (i.e., ≤ 16,950 mg, > 16,950 mg).

The Chi-square test was used to compare the proportions of categorical variables among high-dose acarbose users, low-dose acarbose users, and non-users. A p-value of <0.05 was considered statistically significant.

Supplemental Table S3. Multivariate adjusted ORs with 95% CIs for RA risk associated with low- or high-dose acarbose use among different subgroups

Acarbose	Subgroup for stratified analysis		Effect modification p-value
	Men (n = 2,266)	Women (n = 5,687)	0.839
Non-user	1	1	
Low-dose user	1.07 (0.53–2.15)	0.99 (0.68–1.45)	
High-dose user	0.52 (0.22–1.20)	0.63 (0.40–0.98)	
	Age ≤ 65 (n = 4,880)	Age > 65 (n = 3,073)	0.268
Non-user	1	1	
Low-dose user	1.15 (0.75–1.76)	0.99 (0.58–1.66)	
High-dose user	0.69 (0.43–1.11)	0.43 (0.21–0.89)	
	Periodontitis (n = 1,470)	No periodontitis (n = 6,483)	0.832
Non-user	1	1	
Low-dose user	0.78 (0.30–2.01)	1.06 (0.74–1.52)	
High-dose user	0.77 (0.31–1.88)	0.55 (0.35–0.86)	
	Sulphonylurea/meglitinid	Sulphonylurea/meglitinide	0.302

	e user (n = 3,446)	non-user (n = 4,507)	
Non-user	1	1	
Low-dose user	1.03 (0.66–1.59)	1.03 (0.61–1.74)	
High-dose user	0.44 (0.26–0.77)	0.79 (0.44–1.42)	
	Metformin user (n = 4,518)	Metformin non-user (n = 3,435)	0.607
Non-user	1	1	
Low-dose user	1.01 (0.70–1.47)	0.60 (0.23–1.54)	
High-dose user	0.52 (0.32–0.87)	0.79 (0.41–1.51)	
	TZD user (n = 614)	TZD non-user (n = 7,339)	0.052
Non-user	1	1	
Low dose user	1.34 (0.51–3.53)	1.00 (0.69–1.44)	
High-dose user	1.01 (0.40–2.57)	0.49 (0.31–0.78)	
	Insulin user (n = 237)	Insulin non-user (n = 7,716)	0.081
Non-user	1	1	
Low dose user	2.11 (0.71–6.26)	0.93 (0.64–1.34)	
High-dose user	0.51 (0.10–2.70)	0.56 (0.37–0.85)	
	Statin user (n = 1,947)	Statin non-user (n = 6,006)	0.111
Non-user	1	1	

Low-dose user	1.21 (0.67–2.20)	0.97 (0.65–1.46)
High-dose user	0.80 (0.43–1.48)	0.44 (0.26–0.76)

Abbreviations: OR, odds ratio; CI, confidence interval; TZD, thiazolidinediones.

Acarbose use was further categorized as low- or high-dose based on the median annual cumulative dose (i.e., ≤ 16,950 mg, > 16,950 mg).

Conditional logistic regression model was applied to calculate the ORs with 95% CIs of RA risk associated with acarbose use after adjusting for the duration of diabetes mellitus (DM), DM end-organ disease, Charlson comorbidity index, geographic region, periodontitis history, sulphonylurea/meglitinide use, metformin use, thiazolidinediones use, insulin use and statin use.

The significance of modification of acarbose associated RA protective effect by gender, age (≤ 65 years, >65 years), periodontitis, sulphonylurea/meglitinide use, meformin use, TZD use, insulin use or statin use was assessed using the Wald test to calculate the p-value of the coefficient associated with the product of the indicators of acarbose use and the subgroup.
